# Evaluation of the diagnostic accuracy of a point-of-care device to measure concentrations of nonesterified fatty acids in serum and whole blood

**DOI:** 10.3168/jdsc.2022-0269

**Published:** 2023-02-09

**Authors:** P.L. Venjakob, L.F. Bretzinger, S. Borchardt, C. Weber, W. Heuwieser

**Affiliations:** 1 Clinic for Animal Reproduction, Faculty of Veterinary Medicine, Freie Universität Berlin, 14163 Berlin, Germany; 2 Clinic for Ruminants, Justus-Liebig-Universität Gießen, Frankfurter Str. 104, 35392 Gießen, Germany; 3 Laboklin GmbH and Co. KG, Laboratory for Clinical Diagnostics, Steubenstraße 4, 97688 Bad Kissingen, Germany

## Abstract

The objective of this study was to compare measurements of nonesterified fatty acids (NEFA) between the gold standard diagnostic laboratory method and a handheld NEFA meter (Qucare Pro meter, DFI Co. Ltd.). Three experiments were conducted to study the usability of the meter. In experiment 1 we compared results of the meter obtained from measurements in serum and whole blood with results of the gold standard method. Based on the results of experiment 1 we compared the results measured by the meter in whole blood with results obtained from the gold standard method on a larger scale, as we wanted to omit the step of centrifugation with the cow-side test. In experiment 3 we determined the influence of ambient temperature on measurements. Overall, blood samples of 231 cows were collected between 14 and 20 d in milk. The Spearman correlation coefficients (ρ) were calculated and Bland-Altman plots were created to compare the accuracy of the NEFA meter with the gold standard. In addition, in experiment 2 receiver operating characteristic (ROC) curve analyses were performed, to define thresholds for the NEFA meter to detect cows with a NEFA concentration above 0.3, 0.4, and 0.7 mEq/L. In experiment 1, there was a high correlation between NEFA concentrations in whole blood and serum determined by the NEFA meter and the gold standard (ρ = 0.90 for measurement in whole blood; ρ = 0.93 for measurement in serum). In experiment 2, the measurement in whole blood with the NEFA meter was compared with the gold standard. Despite a lower correlation (ρ = 0.79) the ROC curve analyses revealed a high specificity and a moderate sensitivity for lower cut-points (i.e., 0.3 and 0.4 mEq/L). The NEFA meter underestimated especially high concentration of >0.7 mEq/L. Considering thresholds of 0.3, 0.4, and 0.7 mEq/L measured by the gold standard test, sensitivity and specificity were 59.1% and 96.7%, 79.0% and 95.4%, and 86.4% and 95.6%, respectively, when using 0.3, 0.3, and 0.4 mEq/L as thresholds for the NEFA meter. Accuracy was 74.1%, 88.3%, and 93.8% for the 3 thresholds tested. Experiment 3 showed that measurements should be conducted at approximately 21°C (ρ = 0.73) as correlations were poor at 6.2°C and 15.1°C (ρ = 0.18 and 0.22, respectively).

## Highlights

• The objective of this study was to validate a cow-side meter to estimate the NEFA concentration in whole blood of dairy cows.
• Despite a high specificity, the device had a moderate sensitivity using low thresholds such as 0.3 and 0.4 mEq/L.
• Using a threshold of 0.7 mEq/L, the meter had a high accuracy.
• The meter underestimates high NEFA concentrations >0.7 mEq/L.
• Results of the meter are temperature dependent; measurements must be conducted at around 21°C.

## Graphical Abstract

**Figure:**
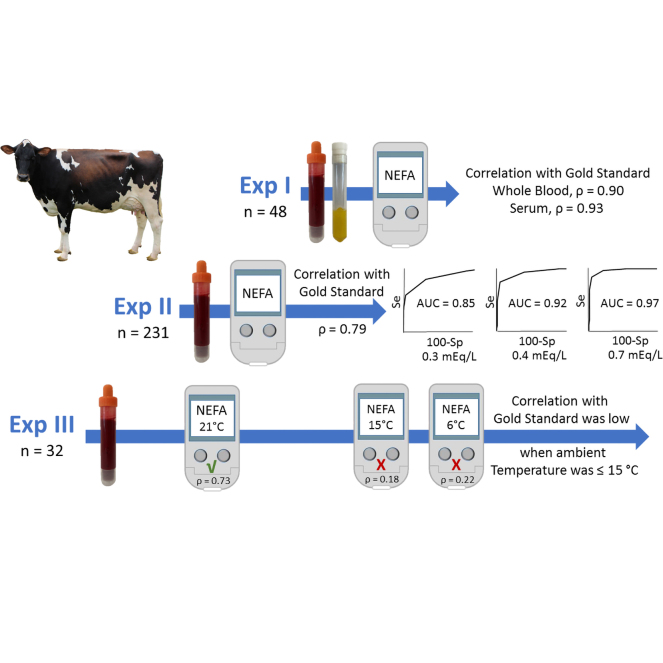
**Summary:** Our objective was to validate a cow-side meter to estimate blood nonesterified fatty acids (NEFA). Specifically, we evaluated whether NEFA concentrations can be measured in whole blood to omit the step of centrifugation on the farm. Therefore, we measured NEFA concentration in lithium heparinized whole blood and serum and compared results to NEFA measurements using the gold standard method. Results obtained from whole blood were comparable with results measured in serum. Using whole blood and thresholds of 0.3 and 0.4 mEq/L, the meter had a high specificity but a moderate sensitivity. Accuracy was high, however, when using 0.7 mEq/L as the threshold. Furthermore we evaluated the effect of ambient temperature on accuracy of the meter. Compared with measurements at an ambient temperature of 21°C, results were poorer at an ambient temperature of 15 and 6°C.

A certain degree of negative energy balance (**NEB**) is a physiological, normal process in the transition period as the cow adjusts to new energy demands due to fetal growth and milk production (Ospina et al., 2010a; Sordillo and Mavangira, 2014). Excessive NEB, however, reflects poor adaptation and results in detrimental effects on health, immune function, production, and reproduction after calving (McArt et al., 2013).

Concentrations of NEFA and BHB are considered biomarkers for NEB during the periparturient period (Abuelo et al., 2020) and their associations with disease incidence, milk production, and reproduction have been studied intensively (Duffield et al., 2009; Ospina et al., 2010b; Chapinal et al., 2011). For both biomarkers, critical thresholds have been determined for the prepartum and postpartum period (McArt et al., 2013).

The quantification of NEFA concentration, however, still requires submission of serum or plasma to specialized laboratories, leading to delays and considerable costs ($11–17; McArt et al., 2013; Abuelo et al., 2020). Most recently, 2 studies evaluated devices that allowed NEFA measurements independent of diagnostic laboratories with promising results. In one study, a small-scale chemistry analyzer (530 × 400 × 500 mm, 15 kg) was used using plasma as sample medium (Abuelo et al., 2020), whereas in the other study a relatively small device (205 × 126 × 110 mm, 800 g) was validated utilizing whole blood (Fukumori et al., 2021). Abuelo et al. (2020) evaluated the accuracy based on a cut-point of 0.3 mEq/L in 150 cows 7 to 13 d before calving while Fukumori et al. (2021) evaluated the accuracy based on cut-points of 0.4 and 0.6 mEq/L over a long period (i.e., 86 d before to 343 d after calving). Correlations between the gold standard laboratory test and the small-scale analyzers (Abuelo et al., 2020: R^2^ = 0.98; Fukumori et al., 2021: R^2^ = 0.90), sensitivity (Abuelo et al., 2020: 94.4%; Fukumori et al., 2021: 93.2%), and specificity (Abuelo et al., 2020: 100%; Fukumori et al., 2021: 99.4%) were high. Disadvantages were the purchase costs, the considerable size or weight of the analyzer, and the necessity to use plasma samples in the older study. The objective of this study was to determine the diagnostic performance of a handheld (130 × 68 × 23 mm, 123 g) NEFA meter (Qucare Pro meter, DFI Co. Ltd.). Specifically, we set out (1) to study the usability of whole blood, (2) to compare the NEFA meter with serum NEFA testing in a specialized laboratory as the gold standard, and (3) to determine the influence of ambient temperature on measurements.

This study was conducted between September 2021 and January 2022. Sample collection was ethically approved as part of a larger field study by the federal authorities of Mecklenburg-Western Pomerania (7221.3–1-047/21) and Thuringia (FUB-21–001). The convenience sample was chosen, based on former studies evaluating the accuracy of handheld devices to evaluate the blood NEFA or BHB concentrations (Bach et al., 2016; Süss et al., 2016; Leal Yepes et al., 2018). Overall, 231 cows from 2 farms milking approximately 720 and 1,000 Holstein Friesian cows were included. Of those, 48, 231, and 32 were included in experiment 1, 2, and 3, respectively. All cows were sampled between 14 and 20 DIM.

Using an 18-gauge, 1.5-inch (3.81 cm) hypodermic needle (Vacuette, Greiner Bio-One GmbH) blood was drawn from the coccygeal vessels. Blood samples were collected into sterile, evacuated whole blood tubes containing lithium heparin as anticoagulant and in serum collection tubes without any anticoagulant (8 mL, Vacuette, Greiner Bio-One GmbH). During the transport to the laboratory of the Clinic of Animal Reproduction samples were stored on ice. Serum samples were allowed to clot for further 2 to 4 h and then centrifuged at approximately 21°C and 4,500 × *g* for 10 min to harvest serum (Heraeus Sepatech Labofuge 200, Heraeus Holding GmbH). Serum was transferred into sterile vials (2 mL, Cryovial, Simport) and one aliquot was frozen and stored at −18°C until NEFA analysis in a commercial laboratory (Laboklin GmbH and Co. KG, Laboratory for Clinical Diagnostics, Bad Kissingen, Germany). Another serum aliquot (experiment 1) and whole blood sample were equilibrated to room temperature (21°C) and NEFA concentration was evaluated using the handheld NEFA meter. All measurements were conducted by the first author according to the guidelines provided by the manufacturer. Briefly, the device was started and a test strip was introduced into the test strip port. Subsequently, the chamber flap was opened and 10 µL of serum or whole blood was pipetted onto the application area. A test strip is composed of a blood spread, a separation, and a reaction layer. The blood separation layer separates plasma from whole blood and the spread layer transports the plasma uniformly to a reaction layer. The assay is based on the acyl-CoA oxidase method (Matsubara et al., 1983). In brief, NEFA are converted to acyl-CoA, AMP, and pyrophosphoric acid by the acyl-CoA synthetase in the presence of CoA and ATP. In a second step, acyl-CoA is oxidized by acyl-CoA oxidase to produce 2,3-*trans*-enoyl CoA and hydrogen peroxide. Hydrogen peroxide causes oxidative condensation of 4-aminoantipyrine and redox dye (i.e., *N*-ethyl-*N*-sulfopropyl-M-toluidine) in the presence of peroxidase and forms a complex with a red-purple color, which is measured at 550 nm wavelength. The same procedure was conducted in experiment 1 for both sample types after changing the settings of the device accordingly. The test results were displayed 120 s after sample application in mEq/L using 0.1 increments. As the gold standard method, serum NEFA concentrations were evaluated according to CLSI guidelines (Clinical and Laboratory Standards Institute, 2012) in a commercial laboratory by photometry using a NEFA test kit (Randox) and concentration was measured using a Cobas 8000 c701 (Roche Diagnostics International AG) in one batch. The interassay coefficients of variation were 1.50% (0.99 mEq/L; n = 5) and 3.81% (1.40 mEq/L; n = 5). The intraassay coefficients of variation were 1.75% (0.99 mEq/L; n = 5) and 1.24 (1.53 mEq/L; n = 5).

In experiment 1, we evaluated whether the NEFA concentration can be measured by the handheld NEFA analyzer in serum as well as in whole blood. For this purpose, whole blood and serum samples from 48 cows were measured using the handheld NEFA meter. To evaluate the accuracy of the NEFA meter serum samples were analyzed by the gold standard as described above. Using SPSS for Windows (version 27.0, IBM Corp.), Spearman correlation coefficients (**ρ**) were calculated between the NEFA concentrations determined by the NEFA meter in whole blood and serum and by the gold standard. A simple linear regression analysis (i.e., Y = a + b∙X) was performed using SPSS where Y was the gold standard, X was the NEFA meter, a was the intercept, and b was the slope of the regression line. We performed a probability-probability plot to make sure that the residuals were normally distributed. Homoscedasticity was assessed by plotting the predicted values and the residuals. Bland-Altman plots (Bland and Altman, 1986) were created using MedCalc (version 19.6.1; MedCalc). The mean serum NEFA concentration determined by the gold standard was 0.60 (±0.33, SD) mEq/L in experiment 1. There was a high correlation between NEFA concentrations in whole blood and serum determined by the NEFA meter and the gold standard (ρ = 0.90 and 0.93 for whole blood and serum, respectively, *P* < 0.001). Bland-Altman plots revealed that the NEFA meter underestimated the NEFA concentration in comparison to the gold standard with a mean bias of −0.26 and −0.33 mEq/L for whole blood and serum, respectively (Figure 1). Upper and lower limits of agreement were 0.02 and −0.54, respectively, for whole blood and −0.03 and −0.59, respectively, for serum. Furthermore, the Bland-Altman plots demonstrated that the NEFA meter underestimates especially high NEFA concentrations of >0.7 mEq/L.

**Figure 1:**
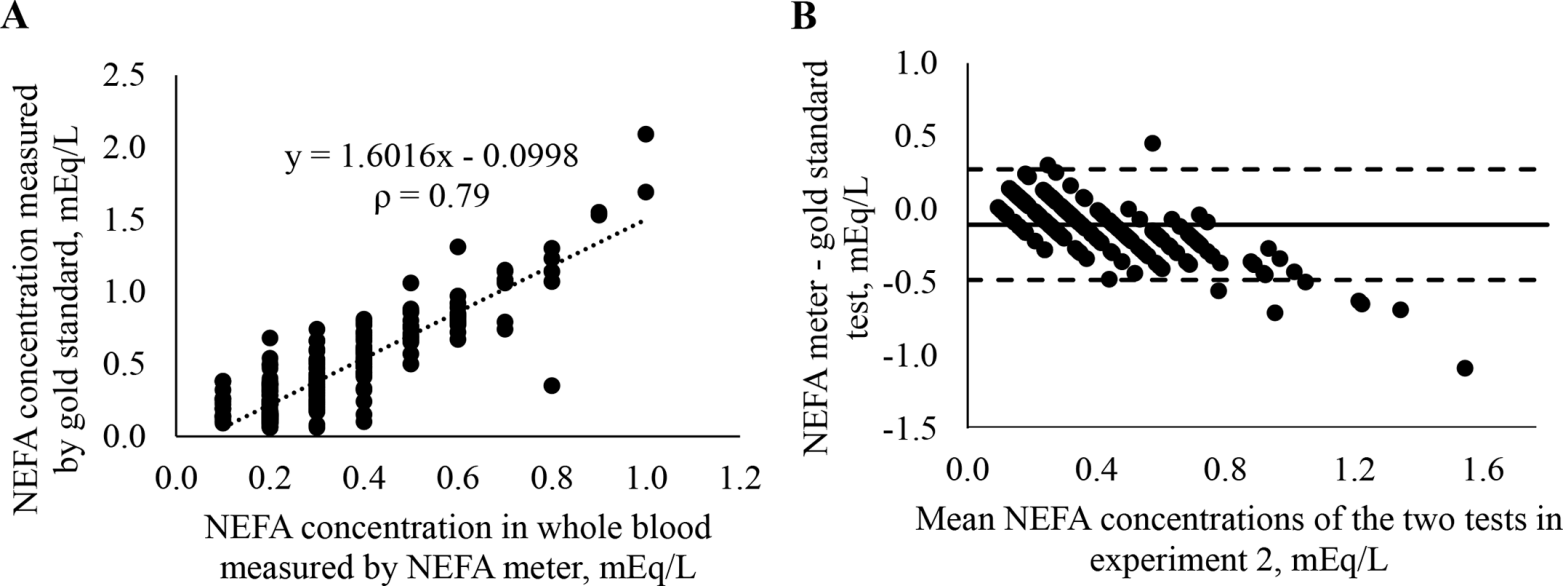
Agreement between the nonesterified fatty acid (NEFA) concentrations determined with the handheld NEFA analyzer in whole blood and serum NEFA concentration determined in a diagnostic laboratory using the gold standard (n = 231). In panel A the correlation between the 2 measurements is displayed (ρ = Spearman correlation coefficient). Panel B displays a Bland-Altman plot of the differences between the NEFA meter and the gold standard against the mean of their results. The solid horizontal line represents the mean bias (−0.11 mEq/L). Horizontal dashed lines represent the 95% CI of agreement.

In experiment 2, we evaluated the accuracy of the NEFA meter, based on a larger number of whole blood samples. A total of 231 whole blood samples were measured using the handheld NEFA analyzer and with the gold standard test as described above. A Bland-Altman plot was created and ρ was calculated between the results obtained from the NEFA meter and the gold standard. Furthermore, 3 different receiver operating characteristic (**ROC**) curve analyses were performed to define thresholds for the NEFA meter to detect cows with a NEFA concentration above 0.3, 0.4, and 0.7 mEq/L, respectively. These thresholds were chosen based on Ospina et al. (2013) and McArt et al. (2013). We calculated the average concentration of NEFA (mean ± SD) and test characteristics [sensitivity (**Se**), specificity (**Sp**), accuracy, positive predictive value, negative predictive value, and area under the curve (**AUC**)] using MedCalc (version 15.6.1; MedCalc). Sensitivity described the probability that a test result, measured with the NEFA meter, correctly indicated a NEFA concentration above the certain threshold measured by the gold standard test. Specificity described the probability that a test result, measured with the NEFA meter, correctly indicated a NEFA concentration below a certain threshold measured by the gold standard test. Accuracy was calculated by dividing the sum of true positive and true negatives by all test results (i.e., TP + TN/TP + TN + FP + FN), where TP = true positive, TN = true negative, FP = false positive, and FN = false negative. Positive predictive value described the probability that a cow had a serum NEFA concentration above the threshold, given that the cow was classified as having NEFA concentrations above the threshold as determined by the NEFA meter. Negative predictive value described the probability that a cow had a serum NEFA concentration below the threshold, given that the cow was classified as having NEFA concentrations below the threshold as determined by the NEFA meter. By plotting the true positive rate against the false positive rate, a ROC curve was generated and the optimal thresholds were assessed. The optimal threshold was defined as the point on the curve with the highest combined Se and Sp. Its deduction was based on the AUC as perfect (AUC = 1), highly accurate (0.9 < AUC < 1), very accurate (0.7 < AUC < 0.9), accurate (0.5 < AUC < 0.7), and noninformative (AUC = 0.5; Swets, 1988). Differences were considered significant at *P* < 0.05. Furthermore, we evaluated the test repeatability for the NEFA meter by reevaluating (n = 10) the NEFA concentration in a sample with a low and a high NEFA concentration (0.10 and 1.69 mEq/L according to the gold standard, respectively). The mean serum NEFA concentration determined by the gold standard was 0.44 (±0.32) mEq/L. According to the gold standard, 136 (58.9%), 102 (44.2%), and 44 (19.1%) of the 231 cows sampled had a serum NEFA concentration above 0.3, 0.4, and 0.7 mEq/L, respectively. Evaluation of the accuracy of the handheld NEFA analyzer using a larger number of whole blood samples confirmed results of experiment 1. The equation of the regression line (y = 1.6016x − 0.0998) shows that the test underestimates high NEFA concentrations compared with the gold standard (Figure 1A). Test characteristics were calculated to determine the ability of the test to discriminate whether NEFA concentrations were above 3 common thresholds (McArt et al., 2013) as determined by the gold standard (i.e., 0.3, 0.4, and 0.7 mEq/L). Considering a threshold of 0.3 mEq/L measured by the gold standard test, Se and Sp were 59.1% and 96.7%, respectively, for a threshold of the NEFA meter of 0.3 mEq/L (AUC: 0.853; *P* < 0.001; Figure 2A). Considering a threshold of 0.4 mEq/L measured by the gold standard test, Se and Sp were 79.0% and 95.4% for a threshold of the NEFA meter of 0.3 mEq/L (AUC: 0.920; *P* < 0.001; Figure 2B). Considering a threshold of 0.7 mEq/L measured by the gold standard test, Se and Sp were 86.4% and 95.6% for a threshold of the NEFA meter of 0.4 mEq/L (AUC: 0.968; *P* < 0.001; Figure 2C). Test characteristics are summarized in Table 1. Evaluating the test repeatability in the sample with a NEFA concentration of 0.1 mEq/L according to the gold standard, the NEFA concentration measured by the NEFA meter was 0.1 mEq/L (±0.00 mEq/L, SD; n = 10, CV = 0.0%). Evaluating the test repeatability in the sample with a NEFA concentration of 1.69 mEq/L according to the gold standard, the NEFA concentration measured by the NEFA meter was 0.85 mEq/L (±0.12 mEq/L, SD; range: 0.70–1.00 mEq/L; n = 10; CV = 14.2%).

**Figure 2:**
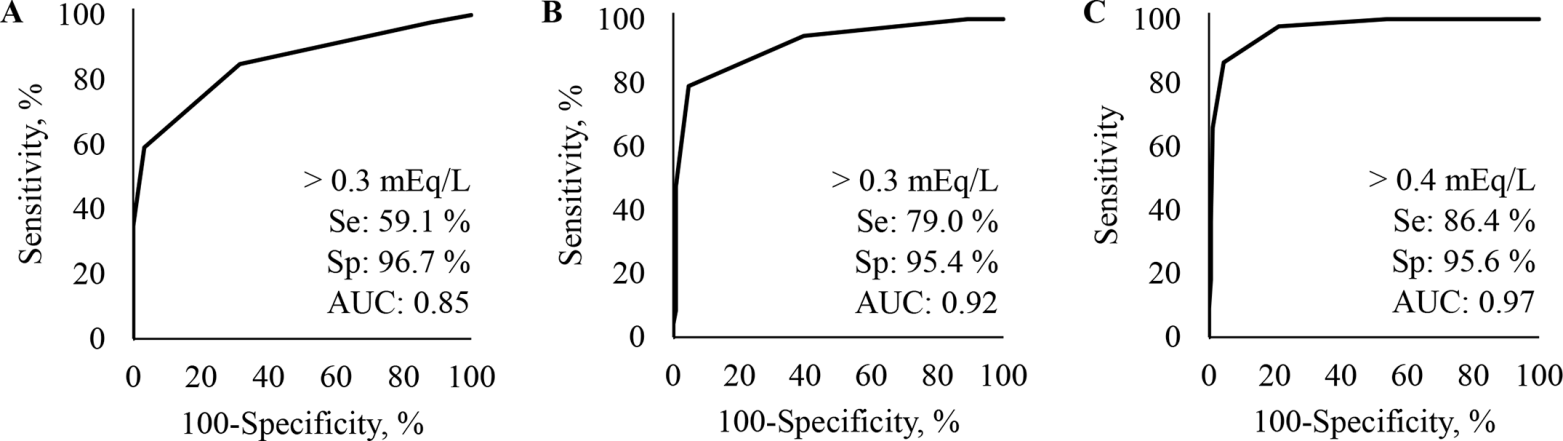
Receiver operating characteristic curve that determines the critical threshold with the best combined sensitivity (Se) and specificity (Sp) to identify cows with a nonesterified fatty acid (NEFA) concentration of >0.3 mEq/L (panel A), >0.4 mEq/L (panel B), and >0.7 mEq/L (panel C). AUC = area under the curve.

**Table 1:** Test characteristics of the handheld nonesterified fatty acid (NEFA) analyzer (Qucare Vet meter, DFI Co. Ltd.) relative to the gold standard measured in a diagnostic laboratory (n = 231) reference test; Randox NEFA kit, Randox

Threshold gold standard,^1^ mEq/L	Threshold NEFA meter,^2^ mEq/L	Sensitivity,^3^ % (95% CI)	Specificity,^4^ % (95% CI)	AUC^5^	Accuracy,^6^ %	Prevalence,^7^ %	PPV,^8^ %	NPV,^9^ %
>0.3	>0.3	59.1 (50.2–67.6)	96.7 (90.8–99.3)	0.853	74.1	60.0	96.4	61.2
>0.4	>0.3	79.0 (69.4–86.6)	95.4 (90.2–98.3)	0.920	88.3	43.2	92.9	85.7
>0.7	>0.4	86.4 (72.6–94.8)	95.6 (91.4–98.1)	0.968	93.8	20.0	83.1	96.6

1 Serum NEFA concentration of 231 cows at 14 to 20 DIM was determined using the Randox test kit and the Cobas 8000 c701 (Roche Diagnostics International AG).

2 Threshold was calculated based on receiver operating characteristic (ROC) curve analysis.

3 The probability of being classified above the threshold using the NEFA meter, given that the serum NEFA concentration was above the threshold determined by the gold standard test.

4 The probability of being classified below the threshold using the NEFA meter, given that the serum NEFA concentration was below the threshold determined by the gold standard test.

5 Area under the curve.

6 Accuracy was calculated by dividing the sum of true positive (TP) and true negatives (TN) by all test results (i.e., TP + TN/TP + TN + FP + FN). FP = false positive; FN = false negative.

7 Proportion of cows being above the threshold using the gold standard.

8 Positive predictive value: the probability that a cow had a serum NEFA concentration above the threshold, given that the cow was classified as having NEFA concentrations above the threshold as determined by the NEFA meter.

9 Negative predictive value: the probability that a cow had a serum NEFA concentration below the threshold, given that the cow was classified as having NEFA concentrations below the threshold as determined by the NEFA meter.

In experiment 3, we investigated whether the ambient temperature affects the accuracy of the handheld NEFA analyzer using whole blood samples. Therefore, the NEFA concentration was measured in 32 cows with the handheld NEFA analyzer at approximately 21°C, 15°C, and 6°C. Immediately after collection of the blood sample, the samples, test strips, handheld NEFA analyzer, and pipette were placed in an office of the farm at approximately 21°C. The samples and the equipment were allowed to equilibrate at this temperature for a period of 45 min. Subsequently all samples were measured as described above. A second and third replicate were conducted at 15°C (in the barn) and 6°C (outside the barn), respectively. For each temperature step, the 45-min equilibration period was adhered to. Simultaneously with each measurement, the surface temperature of the handheld NEFA analyzer was evaluated using an infrared thermometer (Fluke 568 IR Thermometer, Fluke Deutschland GmbH). To compare the performance of the test at different ambient temperatures, ρ was calculated between the NEFA concentrations determined by the NEFA meter in whole blood at 3 different ambient temperatures and the serum NEFA concentration determined by the gold standard. In experiment 3 (NEFA concentration 0.41 ± 0.28 mEq/L) blood samples were first analyzed at ambient temperature. Spearman correlation coefficient between measurement with the handheld NEFA analyzer at 21.2 ± 0.3°C and the gold standard was 0.73 (*P* < 0.001). In a second and third step, the same samples were measured by the NEFA meter at 15.1 ± 0.5°C and 6.2 ± 0.3°C. Spearman correlation coefficient between measurement with the NEFA meter and the gold standard was 0.22 (*P* = 0.237) and 0.18 (*P* = 0.329), respectively.

The objective of this study was to determine the diagnostic performance of a handheld NEFA meter for on-farm use, considering the usability of whole blood and the influence of ambient temperature. Prepartum elevated NEFA concentrations have been associated with a greater risk of abomasal displacement, hyperketonemia, reproductive tract disorders, and culling (Ospina et al., 2010a; Chapinal et al., 2011; Seifi et al., 2011). An accurate, rapid, and inexpensive tool to screen for high NEFA concentrations before calving on site would allow for early identification of cows at risk for secondary diseases. In experiment 1, we showed that not only serum (ρ = 0.93) but also whole blood (ρ = 0.90) can be used as sample medium for the NEFA meter. There was, however, a negative mean bias of −0.33 and −0.26 mEq/L for serum and whole blood, respectively, indicating that the NEFA concentrations were underestimated with the NEFA meter. This underestimation increased with higher NEFA concentrations (Figure 1B). A negative but smaller bias (0.02 mEq/L) was also shown in 2 recent studies evaluating small-scale chemistry analyzers (Abuelo et al., 2020; Fukumori et al., 2021).

Whole blood as sample material is clearly advantageous because no additional equipment and time are needed to separate plasma or serum. The agreement of measurements conducted in serum and whole blood in experiment 1 encouraged us to conduct a second experiment with a larger sample size (n = 231) comparing measurements in heparinized whole blood with the gold standard test. The Spearman correlation coefficient (ρ = 0.79) and the negative bias (−0.11 mEq/L) were lower than in experiment 1. For commercially available BHB meters (Bach et al., 2016), higher ρ (0.96–0.99) were determined. Mean biases (0.08–0.34 mmol/L), however, were also higher relative to the average concentration. The Bland-Altman plot illustrates that the NEFA meter underestimates high NEFA concentrations (i.e., >0.7 mEq/L) compared with the gold standard test, which was also true in the previous studies. Fukumori et al. (2021) speculated that the underestimation could be explained by insufficient influx of oxygen into the reaction chamber to entirely oxidize the NEFA at high concentrations. The authors argued that cows with very high NEFA concentrations were still correctly classified by the tested device as any NEFA concentration over 0.4 to 0.6 mEq/L is classified as high.

We calculated test characteristics for 3 thresholds (i.e., 0.3, 0.4, and 0.7 mEq/L) to address the prepartum (0.3 to 0.5 mEq/L) and postpartum (0.7 to 1.0 mEq/L) NEFA thresholds previously shown to be associated with negative downstream outcomes (McArt et al., 2013; Ospina et al., 2013).

The sensitivities calculated for the 2 lower thresholds in our study were considerably lower than the 94.4% sensitivity described for a small-scale analyzer (Abuelo et al., 2020). This is probably because plasma as a test medium was used both in the small-scale analyzer and the gold standard test. In our study, however, we used whole blood with the NEFA meter and serum for the gold standard test. The different test media might have contributed to the lower accuracy. Also, the manual application of a 10-μL volume with a pipette onto the reaction chamber of the NEFA test strips can have introduced more variability compared with the automatic pipetting of the small-scale analyzer utilized by Abuelo et al. (2020) or placing a 80-μL droplet on the cartridge (Fukumori et al., 2021). The sensitivity calculated for the 0.7 mEq/L threshold of 86.4% is similar to the sensitivity of 87.9% described for a 0.6 mEq/L threshold by Fukumori et al. (2021). The reaction time to reach 0.6 mEq/L was 7 min, whereas the NEFA meter in our study was preset by the manufacturer at only 2 min for all thresholds.

An accurate, rapid, and inexpensive device to screen cows for elevated NEFA concentrations on site could help implement treatment protocols based on the results obtained at specific days before or after calving to avoid blanket treatments that could incur costs without benefits. A positive predictive value of above 90% indicates that there would be relatively few errors of falsely classifying cows as having elevated NEFA concentrations. Due to the relatively high specificity, the NEFA meter might be useful to avoid a costly intervention. Conversely, the moderate sensitivity at the 0.4 mEq/L threshold indicates a 21.0% chance that a cow with increased NEFA concentrations would be missed and a possible beneficial treatment not conducted.

As in the 2 other studies (Abuelo et al., 2020; Fukumori et al., 2021), the NEFA measurements were conducted at room temperature in experiments 1 and 2. However, the validations of a point-of-care device should be performed in the setting where it is intended to be applied (Abuelo and Alves-Nores, 2016). Point-of-care devices for dairy cows could be exposed to lower ambient temperatures when applied cow-side. For BHB meters it has been demonstrated that the temperature of the tested sample influenced the measurements (Iwersen et al., 2013). For NEFA measurements in dairy cattle influence of temperature has not been studied yet. As multiple enzymatic reactions take place in the NEFA strips we hypothesized that the accuracy of NEFA measurements would be affected by lower temperatures. To test this assumption the NEFA meter, test strips, and whole blood samples were equilibrated to 21°C, 15°C, and 6°C for 45 min before the measurement in experiment 3. Our data clearly demonstrate that correlation between the NEFA meter and the gold standard test was poor at 15°C (ρ = 0.22) and 6°C (ρ = 0.18). Therefore, on-farm measurements should not be conducted in the barn with lower ambient temperatures. To prevent such errors, the current version of the NEFA meter has a built-in operating condition of 18 to 30°C outside, when an error message is displayed and measurements cannot be conducted.

Previous studies have also validated relatively small NEFA testing devices using plasma (Abuelo et al., 2020: 15 kg) or whole blood (Fukumori et al., 2021: 800 g). To the best of our knowledge, no study has evaluated a true handheld NEFA meter, which can be used with whole blood on-farm. The present study provides evidence that depending on the threshold used, the evaluated handheld NEFA meter might be a useful tool to identify cows with elevated NEFA concentrations cow-side. We caution the suboptimal sensitivity at lower thresholds and the temperature dependence. The negative bias of 0.11 mEq/L is a result of underestimation of NEFA concentrations evaluated by the NEFA meter. Figure 1B shows that the bias originates especially but not exclusively from samples with a high NEFA concentration (>0.7 mEq/L). The NEFA meter, however, has a high Sp and a moderate Se for lower thresholds (i.e., 0.3 and 0.4 mEq/L) and a high Sp and Se when using 0.7 mEq/L as threshold. The bias, however, shows that the NEFA meter cannot be used to evaluate the NEFA concentration on a continuous scale when evaluating high NEFA concentrations. The tested NEFA meter is the first true handheld device that allows rapid on-farm measurements of NEFA concentrations using whole blood as sample medium. The test strips for the NEFA meter will be available for approximately $5. For the 2 NEFA devices validated previously information on costs per unit are not available. Compared with the costs and inconvenience of a diagnostic laboratory test the NEFA meter might be useful for on-farm NEB monitoring, depending on the threshold. Further research is warranted to evaluate (1) whether on-farm NEFA assessment is helpful to identify cows at risk for secondary diseases and (2) if the composition of fatty acids may affect the meter's performance considering also prepartum samples.
